# Prognostic factors in patients with thymoma who underwent surgery

**DOI:** 10.1186/s12957-023-03068-9

**Published:** 2023-07-11

**Authors:** Yu-Gang Jiang, Mu-Yuan Ma, Jia-Jun Wu, Rong Ma, Xue-Hong Bai, Ren Zhao, Jin-Xi He, Yan-Yang Wang

**Affiliations:** 1 Department of Radiation Oncology, General Hospital of Ningxia Medical University, Yinchuan, 750004 Ningxia China; 2 Graduate School, Ningxia Medical University, Yinchuan, 750004 Ningxia China; 3 Department of Surgical Oncology, General Hospital of Ningxia Medical University, Yinchuan, 750004 Ningxia China; 4 Institute of Medical Sciences, General Hospital of Ningxia Medical University, Yinchuan, 750004 Ningxia China; 5 Cancer Institute, Ningxia Medical University, Yinchuan, 750004 Ningxia China; 6 Department of Thoracic Surgery, General Hospital of Ningxia Medical University, Yinchuan, 750004 Ningxia China

**Keywords:** Thymoma, Surgery, Neutrophils, Smoke, Prognosis

## Abstract

**Purpose:**

Thymoma is the most common primary tumor in the anterior mediastinum. The prognostic factors of patients with thymoma still need to be clarified. In this study, we aimed to investigate the prognostic factors of patients with thymoma who received radical resection and establish the nomogram to predict the prognosis of these patients.

**Materials and methods:**

Patients who underwent radical resection for thymoma with complete follow-up data between 2005 and 2021 were enrolled. Their clinicopathological characteristics and treatment methods were retrospectively analyzed. Progression-free survival (PFS) and overall survival (OS) were estimated using the Kaplan–Meier method and compared by the log-rank test. Univariate and multivariate Cox proportional hazards regression analyses were performed to identify the independent prognostic factors. According to the results of the univariate analysis in the Cox regression model, the predictive nomograms were created.

**Results:**

A total of 137 patients with thymoma were enrolled. With a median follow-up of 52 months, the 5-year and 10-year PFS rates were 79.5% and 68.1%, respectively. The 5-year and 10-year OS rates were 88.4% and 73.1%, respectively. Smoking status (*P* = 0.022) and tumor size (*P* = 0.039) were identified as independent prognostic factors for PFS. Multivariate analysis showed that a high level of neutrophils (*P* = 0.040) was independently associated with OS. The nomogram showed that the World Health Organization (WHO) histological classification contributed more to the risk of recurrence than other factors. Neutrophil count was the most important predictor of OS in patients with thymoma.

**Conclusion:**

Smoking status and tumor size are risk factors for PFS in patients with thymoma. A high level of neutrophils is an independent prognostic factor for OS. The nomograms developed in this study accurately predict PFS and OS rates at 5 and 10 years in patients with thymoma based on individual characteristics.

## Introduction

Thymoma is the most common primary tumor in the anterior mediastinum, although the incidence is generally low [1–3]. Moreover, thymoma is associated with a variety of autoimmune diseases, such as hyperthyroidism, pure red cell aplastic anemia, myasthenia gravis (MG), and endocrine disorders [4]. Treatment options for thymoma include surgery, radiotherapy, and systemic therapy. Surgery is the main treatment method for thymoma [5, 6]. Prognostic factors of postoperative thymoma patients include age, tumor size, World Health Organization (WHO) histological classification, paraneoplastic syndrome, great vessel invasion, Masaoka-Koga stage, TNM stage, and completeness of resection [7–13]. However, all these identified factors are not sufficient to explain the prognosis of patients with thymoma with large heterogeneous features. Therefore, the clinical features and prognostic factors of patients with thymoma still need to be clarified. In this study, we analyzed several indicators after surgical resection of thymoma to determine important prognostic factors. At the same time, the predictive nomogram models were established based on the risk factors of thymoma patients, and the performance of nomogram was measured. We believe that these results will improve our understanding of thymoma and facilitate the development of individualized treatment and optimal follow-up strategies.

## Materials and methods

### Study design and patient population

Patients who underwent radical resection for thymoma at our hospital with complete follow-up and histopathological data from 2005 to 2021 were enrolled and analyzed retrospectively. Patients were excluded according to the following criteria: (i) received neoadjuvant chemotherapy or radiotherapy; (ii) had recently received continuous steroid therapy, active infection, or other bone marrow disease before surgery; and (iii) had a history of other types of cancer within 1 year. The following data were collected from the electronic medical record system: age, gender, presence of autoimmune disease, surgical approach, extent of resection, adjuvant therapy, tumor size, tumor histology according to WHO current classification, and Masaoka-Koga stage. Pre-treatment neutrophil and lymphocyte counts were obtained from the routine blood test within 1 week before surgery. The research protocol was approved by Ningxia Medical University General Hospital (KYLL-2022–0013). Patient consent was waived due to the retrospective study design.

### Treatment methods

The surgery was performed by experienced surgeons, using median sternotomy, muscle-preserving thoracotomy, or video-assisted thoracoscopic surgery (VATS), depending on the tumor size, location, and extension of the thymoma. The resection status was classified as R0 (no residual tumor), R1 (microscopic residual tumor), and R2 (macroscopic residual tumor) according to the pathology of the specimen and the surgical findings. Postoperative treatment options were selected based on resection status, histology, and Masaoka-Koga stage. Patients with type B2 histology and above, non-R0 resection, and stage II and above were considered for radiotherapy. Patients with thymic carcinoma (Tc), R2 resection, and stage IV were recommended for chemotherapy. The most commonly used chemotherapy regimens were CAP (cisplatin, doxorubicin, cyclophosphamide) and TP (paclitaxel and cisplatin). Adjuvant radiotherapy was given 4 to 6 weeks after surgery. The dose range of adjuvant radiotherapy was 45 to 60 Gy. The target mainly covered the postoperative tumor bed.

### Follow-up

Follow-up started from the date of completion of the surgery with or without adjuvant therapy and lasted until the patient’s last follow-up or death. Patients were followed up every 3 months in the first year, every 6 months in the following 2 years, and annually thereafter. Follow-up visits included examination of symptoms, chest CT, and blood tests. Patients with MG also underwent neurological monitoring, including clinical evaluation and laboratory tests. Recurrence was defined as histologically confirmed disease recurrence or recurrence that appeared on radiological imaging and response to treatment. Progression-free survival (PFS) is defined as the time from the date of the surgery to the recurrence or the last follow-up. Overall survival (OS) is defined as the time from the date of the surgery to death from any cause or the last follow-up.

### Statistical analysis

The clinical and demographic characteristics of the enrolled patients were descriptively analyzed, and the median and range of continuous variables, as well as the absolute value and relative frequency of categorical variables, were analyzed. The optimal cutoff value of neutrophils or neutrophil to lymphocyte ratio (NLR) to predict survival was determined using receiver operating characteristic (ROC) curve analysis based on the maximum Youden index. Survival curves were plotted using the Kaplan–Meier method, and log-rank tests were performed to determine statistical significance. Univariate and multivariate Cox proportional hazard regression models were used to analyze the risk factors of PFS and OS. *P* < 0.05 was statistically significant. All statistical analyses were performed using SPSS (version 27.0 SPSS Inc., Chicago, IL, USA).

Based on the clinicopathological variables obtained from univariate Cox regression, we created the nomogram, which was completed using R 4.1 and the rms installation package. The concordance index (C-index) value and calibration curve were used to evaluate the performance of the nomogram. Bootstraps with 1000 resamples were used for these activities [14]. A two-sided *P* < 0.05 was the threshold of significance.

## Results

### Patients’ characteristics

**Fig. 1:**
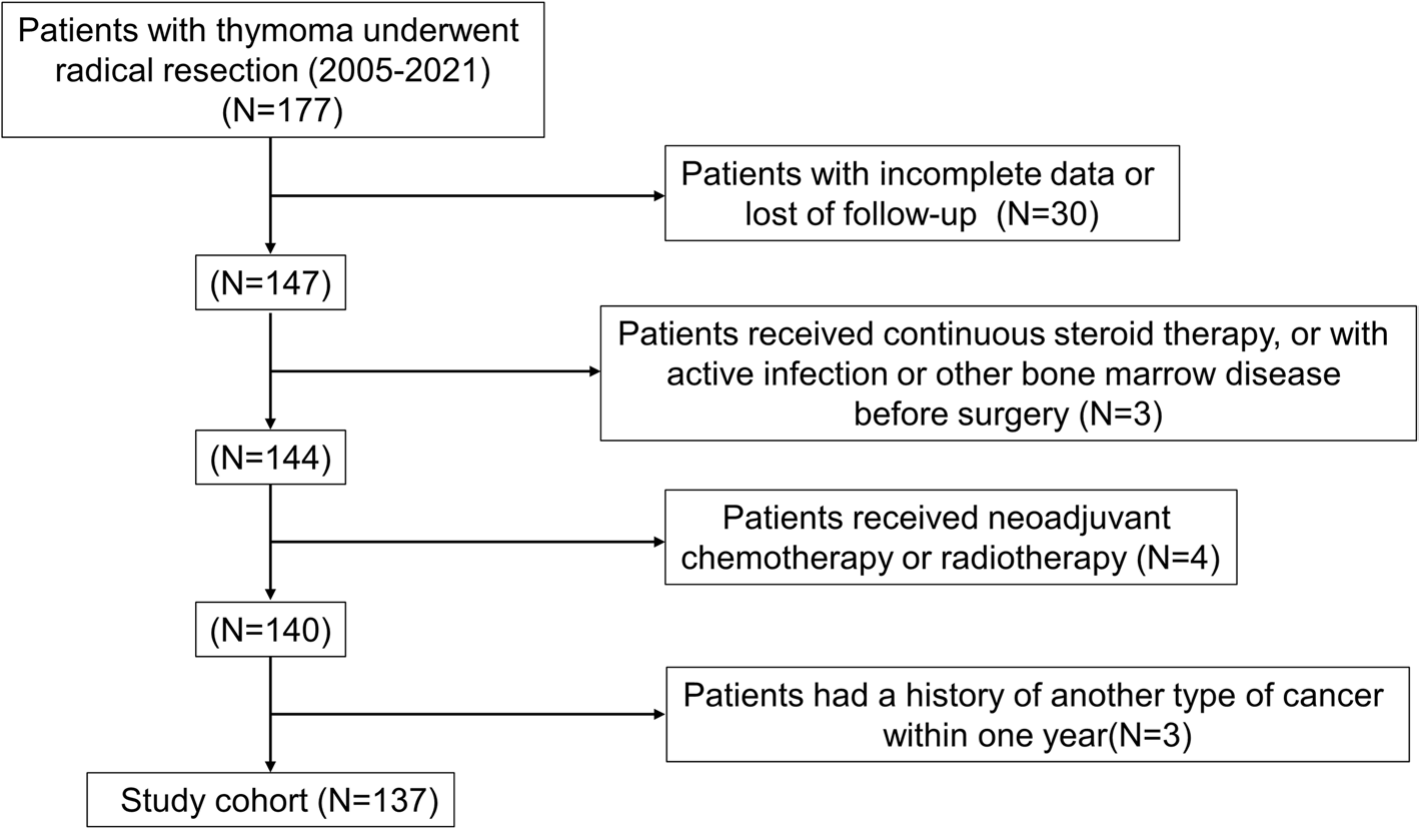
Flow diagram of patient data

A total of 137 patients were enrolled in this retrospective study (Fig. 1). The clinicopathological characteristics of the enrolled patients are shown in Table 1. The median follow-up time was 52 months (2–208 months). At the time of the last follow-up, local recurrence occurred in 17 patients and distant metastasis in 8 patients. The 5-year and 10-year PFS rates were 79.5% and 68.1%, respectively. In addition, 14 patients died during the follow-up period. One patient died of hemoptysis, two patients died of heart failure, and one patient died for unknown reasons with the last follow-up CT scan negative for recurrence. Other patients died of recurrence or metastasis of thymoma. The 5-year and 10-year OS rates are 88.4% and 73.1%, respectively.

**Table 1:** Patients’ characteristics

Characteristic	Number	Percent
Total patients	137	100
Age (years)		
Median	57	
Range	29–87	
Gender		
Male	64	46.7
Female	73	53.3
KPS		
≥ 80	128	93.5
< 80	9	6.5
Smoking status		
Current	37	27.0
Former/never	100	73.0
Myasthenia gravis		
Yes	36	26.3
No	101	73.7
Surgical approach		
Open	56	40.9
VATS	81	59.1
Tumor size		
≥ 6 cm	54	39.4
< 6 cm	83	60.6
Pericardium invasion		
Yes	39	28.4
No	98	71.6
Lung invasion		
Yes	34	24.8
No	103	75.2
Resection status		
R0	85	62.0
Non-R0	52	38.0
WHO classification		
A	7	5.1
AB	33	24.1
B1	33	24.1
B2	37	27.0
B3	14	10.2
Thymic carcinoma	13	9.5
Masaoka-Koga stage		
I	70	51.1
II	12	8.8
III	37	27.0
IV	18	13.1
Ki-67		
≥ 60%	85	62.0
< 60%	52	38.0
Neutrophil		
≥ 3.62	57	41.6
< 3.62	80	58.4
NLR		
≥ 2.85	25	18.2
< 2.85	112	81.8
Adjuvant radiotherapy		
Yes	60	43.8
No	77	56.2
Adjuvant chemotherapy		
Yes	27	19.7
No	110	80.3

*Abbreviations*: *KPS* Karnofsky Performance Status Scale, *VATS* video-assisted thoracic surgery, *WHO* World Health Organization, *NLR* neutrophil-to-lymphocyte ratio

### Univariate and multivariate analysis of PFS and OS

**Fig. 2:**
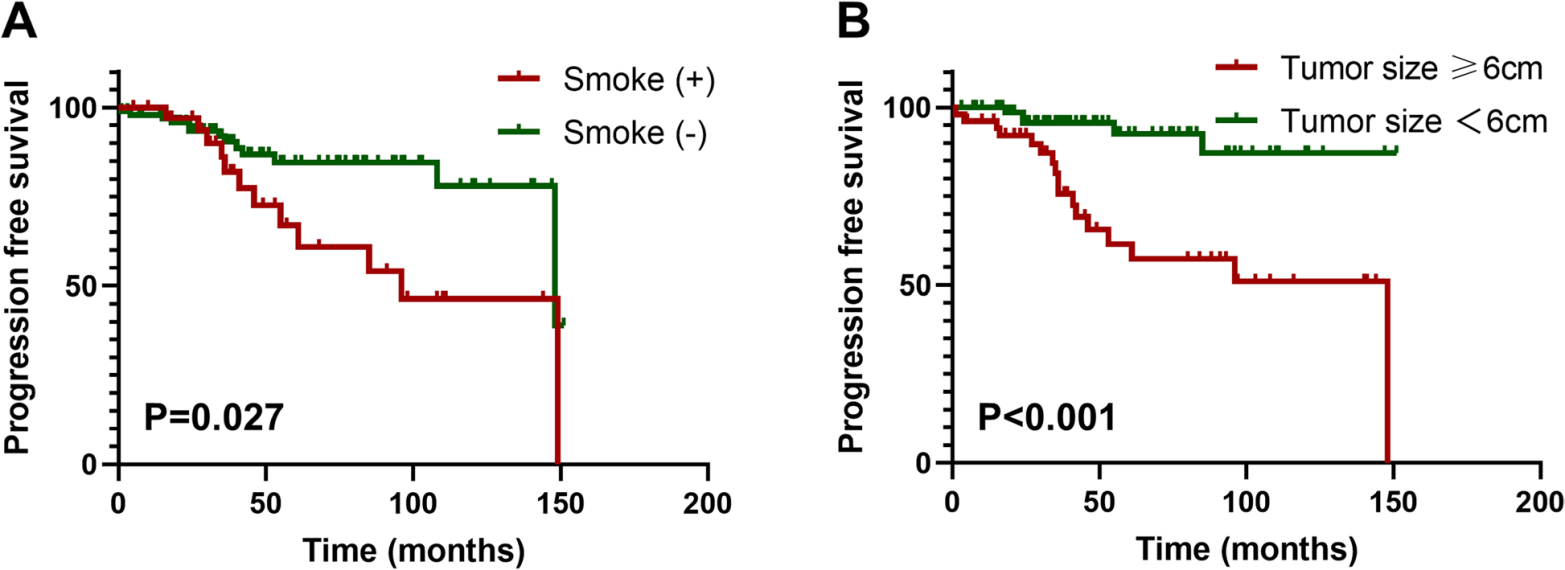
Progression-free Kaplan–Meier survival curves according to smoking status (**A**) and tumor size (**B**)

**Fig. 3:**
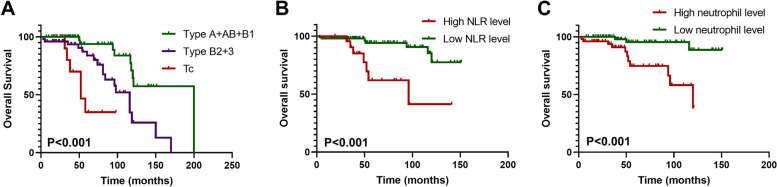
Overall Kaplan–Meier survival curves according to the World Health Organization (WHO) histological classification (**A**), neutrophil-to-lymphocyte ratio (NLR) level (**B**), and neutrophil counts (**C**)

Univariate and multivariate Cox proportional hazard regression models were used to analyze the risk factors of PFS and OS in patients with thymoma. Univariate analysis showed that smoking status, surgical approach, tumor size, resection status, WHO histology classification, Masaoka-Koga stage, adjuvant radiotherapy, and adjuvant chemotherapy were significantly associated with PFS. These factors were included in the multivariate analysis. The results showed that smoking status (*P* = 0.022) and tumor size (*P* = 0.039) were independent risk factors for PFS (Table 2 and Fig. 2). Subsequent univariate analysis showed that the risk factors for OS were WHO classification, neutrophils, and NLR. After multivariate analysis, only neutrophils (*P* = 0.040) remained an independent prognostic factor of OS (Table 3 and Fig. 3).

**Table 2:** Univariate and multivariate Cox regression analyses estimating the risk factors of PFS in thymoma patients

Variable	Univariate analyses			Multivariate analyses		
	HR	95% CI	*P* value	HR	95% CI	*P* value
Age	1.153	0.474 ~ 2.807	0.754			
Gender	1.936	0.853 ~ 4.391	0.114			
KPS	1.647	0.736 ~ 3.681	0.224			
Smoking	2.376	1.080 ~ 5.227	0.031	2.989	1.142 ~ 7.823	0.026
Myasthenia gravis	1.043	0.448 ~ 2.429	0.922			
Surgical approach	3.426	1.348 ~ 8.704	0.01	0.838	0.171 ~ 4.099	0.827
Tumor size	5.597	2.062 ~ 15.193	0.001	4.871	1.166 ~ 20.352	0.030
Resection status	7.941	2.942 ~ 21.432	< 0.001	0.888	0.108 ~ 7.289	0.912
WHO classification	3.595	2.010 ~ 6.428	< 0.001	2.333	0.903 ~ 6.025	0.080
Masaoka-Koga stage	10.268	3.486 ~ 30.250	< 0.001	2.603	0.240 ~ 28.231	0.432
Ki-67	1.240	0.488 ~ 3.151	0.651			
Neutrophil	1.672	0.704 ~ 3.968	0.244			
NLR	1.841	0.709 ~ 4.777	0.210			
Adjuvant radiotherapy	6.094	2.267 ~ 16.379	< 0.001	2.253	0.572 ~ 8.876	0.246
Adjuvant chemotherapy	7.407	3.223 ~ 17.024	< 0.001	2.631	0.688 ~ 10.056	0.157

*Abbreviations*: *PFS* progression-free survival, *HR* hazard ratio, *CI* confidence interval, *KPS* Karnofsky Performance Status Scale, *WHO* World Health Organization, *NLR* neutrophil-to-lymphocyte ratio

**Table 3:** Univariate and multivariate Cox regression analyses estimating the risk factors of OS in thymoma patients

Variable	Univariate analyses			Multivariate analyses		
	HR	95% CI	*P* value	HR	95% CI	*P* value
Age	2.971	0.976 ~ 7.982	0.056			
Gender	1.462	0.506 ~ 4.220	0.483			
KPS	1.400	0.484 ~ 4.048	0.535			
Smoking	1.921	0.664 ~ 5.555	0.228			
Myasthenia gravis	2.162	0.757 ~ 6.181	0.15			
Surgical approach	1.214	0.418 ~ 3.525	0.722			
Tumor size	1.750	0.606 ~ 5.054	0.301			
Resection status	1.196	0.415 ~ 3.250	0.740			
WHO classification	2.277	1.040 ~ 4.987	0.040	1.906	0.893 ~ 4.069	0.096
Masaoka-Koga stage	2.031	0.704 ~ 5.858	0.190			
Ki-67	1.228	0.329 ~ 4.577	0.760			
Neutrophil	7.179	1.958 ~ 25.961	0.003	5.000	1.214 ~ 20.586	0.026
NLR	5.646	1.932 ~ 16.504	0.002	2.499	0.727 ~ 8.590	0.146
Adjuvant radiotherapy	0.654	0.219 ~ 1.959	0.448			
Adjuvant chemotherapy	2.256	0.780 ~ 6.526	0.133			

*Abbreviations*: *OS* overall survival, *HR* hazard ratio, *CI* confidence interval, *KPS* Karnofsky Performance Status Scale, *WHO* World Health Organization, *NLR* neutrophil-to-lymphocyte ratio

### The nomogram prediction model of PFS and OS

**Fig. 4:**
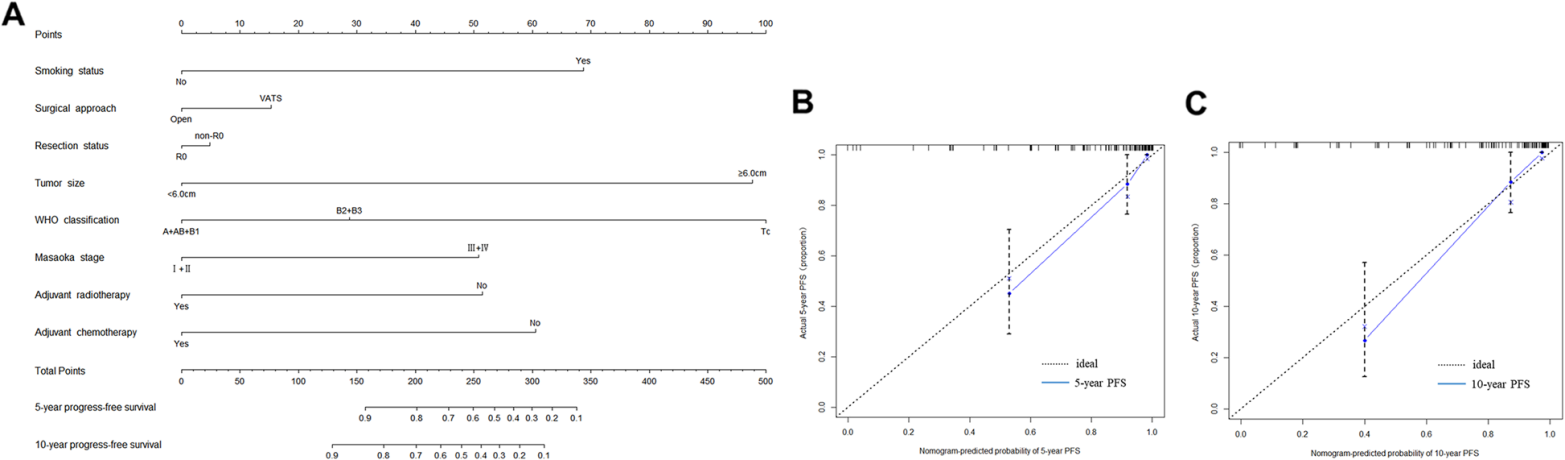
Nomogram for the prediction of 5- and 10-year progression-free survival (PFS) (**A**). Calibration curves of the nomogram-predicted 5-year (**B**) and 10-year PFS (**C**) in the training cohort

**Fig. 5:**
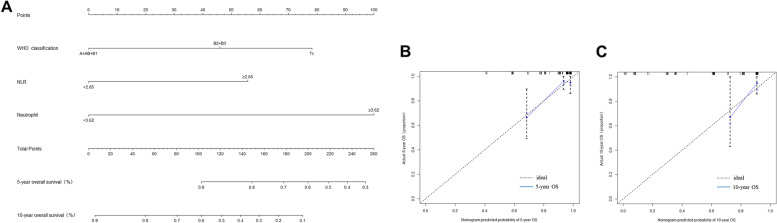
Nomogram for the prediction of 5- and 10-year overall survival (OS) (**A**). Calibration curves of the nomogram-predicted 5-year (**B**) and 10-year (**C**) OS in the training cohort

According to the results of the univariate analysis of the Cox regression model, 8 risk factors were selected to establish a nomogram to predict 5-year and 10-year PFS of thymoma patients, and 3 risk factors were selected to establish a nomogram to predict 5-year and 10-year OS. Nomogram showed that WHO classification contributed more to the risk of recurrence than other factors (Fig. 4). Neutrophil count was the most important predictor of OS in patients with thymoma (Fig. 5). Five-year PFS, OS and 10-year PFS, and OS could be estimated according to the total scores of each factor. Ninety-seven patients were enrolled in the training cohort, and other patients were enrolled in the validation cohort. The C-index for predicting PFS was 0.899 (95% CI, 0.842 ~ 0.956). The C-index for predicting OS was 0.804 (95% CI, 0.700 ~ 0.908). The calibration curves of 5-year and 10-year survival probabilities showed good agreement between the predictions of the nomogram and the actual observations (Figs. 4 and 5).

## Discussion

Thymic epithelial tumors are relatively rare, with an incidence of 3.2 per 1 million people [1–3]. Approximately 80% of thymic epithelial tumors are thymomas [15]. Surgery is the main treatment for thymoma [5, 6]. In this study, we explored patients with thymoma who underwent surgery at our hospital over a 16-year period and analyzed prognostic factors for PFS and OS of these patients. We found that smoking status and tumor size were the major factors affecting PFS. In the subsequent analysis, we revealed that a high level of pretreatment neutrophils was an independent prognostic factor for OS.

The association between smoking and thymoma has been evaluated in several previous studies. In a European multicenter case–control study, Eriksson et al. [16] found that smoking and high spirits intake were risk factors for the development of thymoma. Yanagiya et al. [17] demonstrated that extrathymic malignancies increased the risk of death in patients with thymoma. History of smoking was a risk factor for postoperative extrathymic malignancies. Furthermore, in a recent study, smoking was identified as a critical factor negatively affecting PFS in patients with thymoma [13]. Our results are consistent with previous studies in which we found that smoking was one of the main factors affecting PFS in patients with thymoma. To avoid missed recurrences, postoperative thymoma patients with a history of smoking should be closely followed up. The maximum diameter of most thymomas can be easily measured and obtained from preoperative CT and magnetic resonance imaging (MRI). Therefore, the prognostic value of tumor size in patients with thymoma has been extensively evaluated in previous studies. Several studies have confirmed that thymic tumor size is an independent predictor of recurrence [18–21]. In the present study, PFS was significantly affected with increasing tumor size, but not OS, suggesting that tumor size is a good predictor of recurrence in patients with thymoma.

Neutrophils are first responders to infection and inflammation and also have a role due to their cancer-promoting properties [22, 23]. Neutrophils and their associated markers have been reported to predict poor prognosis in patients with various types of cancer [24]. Recently, thymoma-neutrophil interactions have also been reported. Thymoma produces cytokine-neutralizing autoantibodies and interleukin 12 (IL-12) autoantibodies, which are involved in the regulation of neutrophil maturation [25]. Okada et al. [26] showed that high neutrophil counts were significantly associated with the recurrence of thymoma. In the present study, a high level of neutrophils was an independent prognostic factor for OS. High neutrophil count deserves further study as a biomarker reflecting the prognosis of thymoma patients.

WHO histological classification [27], Masaoka-Koga system stage, and TNM stage are three important prognostic factors of patients with thymoma. In our study, WHO histological classification was associated with OS in univariate analysis, but its independent prognostic effect could not be maintained after adjusting for various multivariates. The Masaoka-Koga system stage was reported to be an important prognostic factor for patients with thymoma and determined the adjuvant treatment options for patients [28]. Due to the overlap between Masaoka-Koga system staging and TNM staging [29], only Masaoka-Koga staging was used in the pathological staging of thymoma in this study. Our results suggest that the Masaoka-Koga system stage was significantly associated with PFS in patients with thymoma; however, this was only found in the univariate analysis. The differences between the results of the current study and those of previous studies are mainly due to the different study populations, the large time span of the included patients, and the changing treatment strategies [30].

Finally, we constructed the nomogram model capable of estimating PFS and OS at 5 and 10 years in patients with thymoma based on the results of univariate Cox regression. The nomogram showed relatively good performance and thus may provide a reliable tool for individual prognostic analysis of thymoma patients. However, prospective studies are needed to further validate the reliability of the nomogram.

There are some limitations in this study. First, this is a retrospective study. Retrospective and nonrandomized properties make selection bias unavoidable. Second, we used cutoff values determined based on ROC curves. This method of obtaining cutoff values has some limitations. Third, we used only baseline values of neutrophils without considering the dynamic changes of this factor. Fourth, only general clinicopathological features were selected to evaluate the prognosis of patients with thymoma. Some details of surgical treatment, such as intraoperative and postoperative complications, can provide more prognostic factors [31] and can be evaluated in further studies. Therefore, in the future, large, prospective, and randomized trials are needed to fully validate the results of this study.

In conclusion, we retrospectively analyzed the prognostic factors of thymoma patients undergoing surgery. The results showed that smoking status and tumor size were the main factors affecting PFS and that baseline high levels of neutrophils were independent prognostic factors for OS. The nomograms developed in this study accurately predict PFS and OS rates at 5 and 10 years in patients with thymoma based on individual characteristics. Our findings may be helpful to determine treatment and follow-up strategies for thymoma patients after radical resection.

## Data Availability

All remaining data are available within the article or available from the corresponding authors upon request.
